# Pharmacological rescue of mitochondrial and neuronal defects in *SPG7* hereditary spastic paraplegia patient neurons using high throughput assays

**DOI:** 10.3389/fnins.2023.1231584

**Published:** 2023-09-12

**Authors:** Gautam Wali, Yan Li, Erandhi Liyanage, Kishore R. Kumar, Margot L. Day, Carolyn M. Sue

**Affiliations:** ^1^Neuroscience Research Australia, Sydney, NSW, Australia; ^2^Kolling Institute for Medical Research, University of Sydney, NSW, Australia; ^3^University of New South Wales, Sydney, NSW, Australia; ^4^Translational Neurogenomics Group, Molecular Medicine Laboratory and Department of Neurology, Concord Repatriation General Hospital, Concord Clinical School, University of Sydney, Concord, NSW, Australia; ^5^Garvan Institute of Medical Research, Darlinghurst, NSW, Australia; ^6^School of Medical Science, Faculty of Medicine and Health, University of Sydney, Sydney, NSW, Australia

**Keywords:** hereditary spastic paraplegia (HSP), mitochondria, induced pluripotent stem (iPS) cell, cortical neurons, high throughput imaging (HTI)

## Abstract

*SPG7* is the most common form of autosomal recessive hereditary spastic paraplegia (HSP). There is a lack of HSP-*SPG7* human neuronal models to understand the disease mechanism and identify new drug treatments. We generated a human neuronal model of HSP-*SPG7* using induced pluripotent stem (iPS) cell technology. We first generated iPS cells from three HSP-*SPG7* patients carrying different disease-causing variants and three healthy controls. The iPS cells were differentiated to form neural progenitor cells (NPCs) and then from NPCs to mature cortical neurons. Mitochondrial and neuronal defects were measured using a high throughout imaging and analysis-based assay in live cells. Our results show that compared to control NPCs, patient NPCs had aberrant mitochondrial morphology with increased mitochondrial size and reduced membrane potential. Patient NPCs develop to form mature cortical neurons with amplified mitochondrial morphology and functional defects along with defects in neuron morphology − reduced neurite complexity and length, reduced synaptic gene, protein expression and activity, reduced viability and increased axonal degeneration. Treatment of patient neurons with Bz-423, a mitochondria permeability pore regulator, restored the mitochondrial and neurite morphological defects and mitochondrial membrane potential back to control neuron levels and rescued the low viability and increased degeneration in patient neurons. This study establishes a direct link between mitochondrial and neuronal defects in HSP-*SPG7* patient neurons. We present a strategy for testing mitochondrial targeting drugs to rescue neuronal defects in HSP-*SPG7* patient neurons.

## Introduction

Hereditary spastic paraplegia (HSP) is an inherited, progressive neurodegenerative disease, causing spasticity in the lower limbs as a consequence of corticospinal tract degeneration. HSP-*SPG7* is the most common form of autosomal recessive HSP (Lange et al., 2022; Méreaux et al., 2022). *SPG7* encoded paraplegin is involved in multiple mitochondrial processes including mitochondrial protein quality surveillance (Casari et al., 1998), mitochondrial biogenesis (Nolden et al., 2005), and regulation of the mitochondrial permeability transition pore (Sambri et al., 2020). *Spg7* knock-out mouse model mimic the clinical feature of HSP-*SPG7* patients (Ferreirinha et al., 2004). Paraplegin-deficient mice showed slow progressive motor impairment with difficulty in maintaining balance on the rotarod associated with distal axonopathy of spinal axons. Mitochondrial morphological abnormalities, i.e., swollen mitochondrial (at 4.5 months) was the first pathological sign observed in the spinal cord axons of paraplegin-deficient mice several months before any evidence of axonal swelling (at 8 months), and degeneration (at 15 months) was detected (Ferreirinha et al., 2004). The mitochondrial phenotype correlated with the onset of motor impairment on the rotarod apparatus at 4 months of age, suggesting that it is the primary cause for axonal dysfunction and that the gait impairment of paraplegin-deficient mice is not directly the result of the loss of axons. Further, intramuscular delivery of paraplegin cDNA *via* adeno-associated viral vectors rescued mitochondrial morphological abnormalities and ameliorated the rotarod performance of paraplegin-deficient mice (Pirozzi et al., 2005), thus identifying mitochondria as a new therapeutic target to develop treatments for HSP-*SPG7*.

In another study, primary neuronal cultures from paraplegin-deficient mice had impaired opening of the mitochondrial permeability transition pore causing dysregulated synaptic activity and impaired synaptic vesicle dynamics leading to ineffective synaptic transmission (Sambri et al., 2020). Pharmacological treatment with Benzodiazepine (Bz) - 423 at low nano molar doses regulated the mitochondrial permeability transition pore opening, normalised synaptic transmission, and rescued motor impairment of the paraplegin-deficient mice. Unfortunately, Bz-423 has off-target effects. It is anti-proliferative and cytotoxic at higher concentrations and is a immunomodulator (Sundberg et al., 2006) making it less desirable to be considered for a therapeutic treatment for *SPG7* patients.

Not much is known about HSP-*SPG7* disease-phenotypes in human cortical neurons. The advent of induced pluripotent stem (iPS) cell technology has opened up the possibility of a scalable source of human cells to produce disease-relevant models to understand the disease-mechanism and identify disease-associated phenotypes that can be used as cellular biomarkers for drug discovery. iPS cells can be readily produced from each patient’s peripheral blood mononuclear cells (PBMCs) or skin fibroblasts, and reliably differentiated into different cells of the central nervous system, including cortical neurons that are degenerated in HSP patients (Wali et al., 2020). Patient-derived iPSCs have been generated for multiple forms of HSP including *SPG4* (Wali et al., 2020), *SPG11* (Pérez-Brangulí et al., 2014), *SPG15* (Denton et al., 2018) and *SPG48* (Denton et al., 2018). These cell models have been able to recapitulate disease-associated phenotypes including reduced axonal transport, and increased axonal swellings, and degeneration. Here we evaluate the disease-associated phenotypes of HSP-*SPG7* using iPS cell technology. We first generated iPS cells from three HSP-*SPG7* patients carrying different pathogenic variants and three healthy controls. iPS cells were differentiated to form neural progenitor cells (NPCs) and then from NPCs to mature cortical neurons.

HSP-*SPG7* patient NPCs showed defects in mitochondrial morphology and function. The mitochondrial in patient NPCs were larger in size and had reduced membrane potential compared to control NPCs. These patient NPCs progressed to form mature neurons with amplified mitochondrial defects, shorter and less complex neurites, downregulated expression of genes related to synaptic function, reduced synaptic activity, reduced viability and increased neurite degeneration. To access the role of mitochondrial in the neuronal phenotypes observed in the patient mature neurons, we treated patient neurons with Bz-423, regulator of the mitochondrial permeability transition pore, as they differentiated from NPCs to mature neurons. Bz-423 treatment restored normal mitochondrial function and rescued neuronal phenotypes, i.e., short and less complex neurites, viability and neurite degeneration in mature neurons, suggesting that mitochondria dysfunction leads to disease-associated phenotypes in HSP-*SPG7* and that mitochondria are a potential therapeutic target. We evaluate mitochondrial and neuronal phenotypes in live neurons using relatively inexpensive live cell dyes (compared to antibodies) and automated high throughput imaging and analysis. This assay enables future drug screening applications to identify a potential drug treatment candidate for HSP-*SPG7*.

## Methods

### Participants

Hereditary spastic paraplegia patients in this study were reviewed and examined by Professor Carolyn Sue and Dr. Kishore Kumar, movement disorder specialists. All patients had a confirmed diagnosis of HSP-*SPG7* on genetic testing. Age, gender, and gene mutation details of the study participants are presented in Table 1. Clinical details of Patient 1 was published previously (identified as Patient 3 in (Wali et al., 2020)) and clinical details of Patients 2 and 3 are presented in Supplementary Table S1. Our study involving human participants was reviewed and approved by Human Research Ethics Committee at Northern Sydney Local Health District Human Research Ethics Committee, Australia (2019/ETH08193) and written informed consent was obtained from all participants.

**Table 1:** Participant details.

Paticipant	Age	Gender	Exon	Mutations
*Controls*				
Control1	53	M		
Control2	51	M		
Control3	47	F		
*SPG7 patients*				
Patient1	62	M	Exon 10	c.1449 + 1G > A
			Exon 11	c.1529C > T
Patient2	52	M	Exon 4	c.415C > T
			Exon 7	c.941 T > A
Patient3	47	M	Intron 16-Exon 17	chr16:g.89623293A > G, c.2182-2A > G
			Exon 11	c.1529C > T

Age, gender and/or mutation details of the study participants.

### Generation of iPS cell lines from PBMCs

#### Blood collection and PBMC isolation

The BD Vacutainer® CPT™ Mononuclear Cell Preparation Tube - Sodium Heparin (Catalog no: 362781, BD Biosciences) is a single tube system for collection of whole blood and separation of peripheral blood mononuclear cells (PBMCs). The tubes contain anticoagulant, FICOLL™ Hypaque™ density fluid and a polyester gel barrier. 4mls of whole blood was collected from each participant. The blood tube was then centrifuged at 1800 relative centrifugal field (RCF) for 15 min at room temperature. After centrifugation, the PBMCs and plasma are separated from red blood cells by the gel barrier. Above the gel barrier, the PBMCs form a layer of cells just below the plasma. The plasma layer was removed and the PBMCs were isolated. PBMCs were washed twice in Phosphate-buffered saline (PBS) buffer +2% Fetal Bovine Serum solution. Finally, PBMCs were counted and frozen down in CryoStor® CS10 freezing media (Catalog no: 07930, Stem cell technologies) and stored in cryo tanks for long term storage (Puleo et al., 2017).

#### Reprogramming of PBMCs to iPS cells

iPS cells were generated by reprogramming PBMCs using the CytoTune®-iPS 2.0 Sendai Reprogramming Kit (Catalog no: A16518, Life Technologies) as per the manufacturer’s manual. Briefly, PBMCs were cultured at a density of at 5 × 10^5^ cells/mL in 6 well plates for 4 days (Day −4 to 0) in PBMC medium, i.e., StemPro™-34 SFM (1X) (Catalog no: 10639011, Thermofisher scientific), supplemented with 2 mM L-Glutamine (Catalog no: 25030081, Thermofisher scientific) and cytokines: 100 ng/mL SCF (C-Kit Ligand) Recombinant Human Protein (Catalog no: PHC2111, Thermofisher scientific), 100 ng/mL FLT3 Ligand Recombinant Human Protein (Catalog no: PHC9414, Thermofisher scientific), 20 ng/mL IL-3 (Catalog no: PHC0034, Thermofisher scientific) and 20 ng/mL IL-6 (Catalog no: PHC0065, Thermofisher scientific). The cytokines should be added on the day of media use. Every other day, half of the medium was replaced with fresh PBMC medium. On Day 0, the cells were transduced with the CytoTune-iPS Sendai reprogramming kit vectors KLF 4, c-MYC, SOX 2 and OCT 3/4. On Day 1, full medium was replaced with fresh PBMC medium to remove the CytoTune™ 2.0 Sendai reprogramming vectors. On Day 3, the transduced cells were plated in a 6 well plate coated with Vitronectin, truncated human recombinant (VTN-N) (Catalog no: A14700, Thermofisher scientific). On Day 4 and 6, full medium was replaced with StemPro™-34 medium without cytokines. On Day 7, the cultured cells were transitioned from StemPro™-34 medium to mTeSR™1 medium (Catalog no: 85850, Stemcell technologies) by replacing half of the StemPro™-34 medium with mTeSR™1 medium. Day 8 to 28, every other day full medium was replaced with mTeSR™1 medium. iPS colonies appeared around Day 18. After 28 days, iPS colonies were transferred to new vitronectin coated plates and immunostaining was performed with pluripotent stem cell markers to confirm their pluripotent identify. Characterization of the iPS cells lines is presented in Supplementary methods and Supplementary Figures S1, S2.

#### Differentiation of iPS to neural progenitors

iPS cells were differentiated into cortical neural progenitor cells (NPCs) using the dual SMAD induction and FGF2 expansion protocol for 25 days (Gantner et al., 2021). First, a 24-well cell culture plate was coated with 15 μg/mL of Poly-L-ornithine solution (Catalog no: P4957, Sigma) at room temperature for 2 h and then with 10 μg/mL of Laminin-mouse (Catalog no: L2020, Sigma) at room temperature for another 2 h. Following plate coating, iPS cells were seeded at a density of 7.125 × 10^5^ cells per well. On Day 0, i.e., the day of iPS cell seeding, the cells were cultured in the cortical neuron base medium with 10 μM Rock inhibitor Y-27632 (Catalog no: 72304, StemCell technologies). From Days 1–10, the cells were cultured in cortical neuron base medium with 100 nM of LDN193189 (2HCl) (Catalog no: 72147, StemCell technologies) and 10 μM of SB431542 (Catalog no: 72232, StemCell technologies). From Days 11–19, the cells were cultured in cortical neuron base medium with 20 ng/mL of FGF2. From Days 20–25, the cells were cultured in cortical neuron base medium only. During this 25-day culture period, the cells were passaged two times, i.e., on Day 11 and Day 20. While passaging, the cortical neuron base medium with 10 μM Rock inhibitor Y-27632 was used. The 10 μM Rock inhibitor Y-27632 was withdrawn after 24 h. On Day 25, the cells were ready for further differentiation into mature cortical neurons or to differentiate them at a later time. The cells were frozen with CryoStor solution (Catalog no: 07930, StemCell technologies) and stored in a cryo tank for long term storage.

Cortical neuron base media comprises of: 48.205% of DMEM/F-12 (Catalog no: 11320033, Gibco), 48.205% of Neurobasal plus media (Catalog no: A3582901, Gibco), 1% of B27 Supplement (Catalog no: 17504044, Gibco), 0.5% of N_2_ Supplement (Catalog no: 17502048, Gibco), 0.5% of ITS-A (Catalog no: 51300044, Gibco), 0.5% of MEM NEAA (Catalog no: 11140050, Gibco), 0.5% of GlutaMax (Catalog no: 35050061, Gibco), 0.5% of Pen/Str (Catalog no: 15140122, Gibco), 0.09% of β-Mercaptoethanol (Catalog no: 21985023, Gibco).

#### Differentiation of neural progenitors to mature cortical neurons

Neural progenitors were differentiated into mature cortical neurons in 96-well (for imaging-based experiments) or 24-well plates (for RNA-Seq and protein extraction) using a modified version of our differentiation protocol (Wali et al., 2023). On Day 0, as described in the “*Differentiation of iPS to neural progenitors”* method section above, the plates were first coated with poly-L-ornithine and laminin. Then, neural progenitors were seeded at a density of 1 × 10^4^ cells per well of a 96-well plate and 1 × 10^5^ cells per well of a 24-well plate. The neural progenitors were seeded in cortical neuron base medium with 10 μM Rock inhibitor. On Days 1 and 2, the cells were cultured in cortical neuron base media. From Days 3 to 10, the cells were cultured in cortical neuron mature medium containing multiple growth factors: 40 ng/mL of both BDNF and GDNF (Catalog no: 78005, 78,058, StemCell technologies), 50 μM of dibutyryl cAMP (Catalog no: 73884, StemCell technologies,), 200 nM of Ascorbic acid (Catalog no: A4403, Sigma), 100 ng/mL of mouse Laminin (Catalog no: L2020, Sigma), and 10 μM of DAPT (Catalog no: D5942, Sigma).

Cortical neuron mature media comprises of: 46.75% of DMEM/F-12 (Catalog no: 11320033, Gibco), 46.75% of Neurobasal plus media (Catalog no: A3582901, Gibco), 2% of B27 Supplement (Catalog no: 17504044, Gibco), 1% of N2 Supplement (Catalog no: 17502048, Gibco), 1% of ITS-A (Catalog no: 51300044, Gibco), 1% of MEM NEAA (Catalog no: 11140050, Gibco), 1% of GlutaMax (Catalog no: 35050061, Gibco), 0.5% of Pen/Str (Catalog no: 15140122, Gibco).

#### Bz-423 drug treatment

A 3 mM Bz-423 stock solution was prepared in DMSO (Catalog no: SML1944-5 mg, Sigma). The stock solution was diluted to a working solution concentration of 150 nM in cell culture media. During the differentiation of neural progenitors to mature cortical neurons, the patient neurons were treated with 150 nM Bz-423 for 7 Days from Day 3 to Day 10.

#### Immunostaining neurons with Tuj1, TBR1 and CTIP2

The Cytofix/Cytoperm™ Fixation/Permeabilization kit (Catalog no: 554714, BD live Sciences-Biosciences) was used to perform the immunofluorescence staining protocol. (a) Media from the 96-well plate was aspirated out. (b) Neurons were fixed using the Cytofix solution for 25 min. (c) Neurons were washed twice using the Cytoperm solution. (d) Neurons were permeabilized and blocked using the Cytoperm solution for 25 min. (e) Neurons were incubated with primary antibodies anti-Tuj1 (Catalog no: ab195879, Abcam) or anti-TBR1 (Catalog no: ab183032, Abcam) or anti-CTIP2 (Catalog no: ab18465, Abcam) for 1 h at a dilution of 1:1000. (f) Neurons were washed twice using the Cytoperm solution. (g) Neurons were incubated with secondary antibodies (Catalog no: A-11012, or A-21471, Invitrogen) at a dilution of 1:500. (h) Neurons were washed twice using the Cytoperm solution. (i) Neurons were labelled with 0.1 μg/mL of Hoechest 33,342 (Catalog no: 62249, Thermo fisher Scientific) for 10 min to identify the nucleus. (j) Neurons were washed twice and maintained in the Cytoperm solution for imaging.

#### Labelling neurons with live cell markers Calcein, TMRM and Hoechst

A cocktail of live cell labelling dyes were used to co-label neurons to identify viable cells (2 μM Calcein, Catalog no: C3100MP, Thermo fisher scientific), mitochondrial (25 nM TMRM, Catalog no, T668, Thermo fisher scientific) and nucleus (0.1 μg/mL Hoechst 33342, Catalog no, 62249, Thermo fisher Scientific). To label live neurons, these dyes were mixed in the cell culture media. Neurons were incubated with the cell culture media and dye cocktail for 30 min at 37°C. The neurons were then washed twice with HBSS solution and maintained in it for imaging. Imaging was performed within 30 min of labelling the cells.

#### Neuron microscope imaging

The images were captured at 20x magnification on the high throughput imaging system, Perkin Elmer Phenix Plus. Images were captured from 3 fluorescence channels: Hoechst (375/456), Calcein/Tuj1 (488/522) and TMRM/TBR1/CTIP2 (561/599). 10 field of views were acquired per well of a 96 well plate. Duplicate wells were imaged per sample.

#### Image processing

Neuron images were processed using the image analysis software Harmony built in with the PhenixPlus, Perkin Elmer high throughput imaging system. First, the nucleus of the neurons was segmented and identified using the “Find Nuclei” building block. The parameters used in the ‘find nuclei’ block were as follows: method, B; common threshold, 0.40; area > 30 μm^2^; splitting coefficient, 7.0; individual threshold, 0.40; contrast >0.10. Then the neurites were identified using the “Find neurites” building block. The parameters used in the ‘find neurites’ block were as follows: smoothing width, 3px; linear window, 11px; contrast, >1; diameter, ≥ 7px, gap closure distance, ≤ 9px; gap closure quality, 1; debarb length ≤ 15px, body thickening, 5 pm and tree length, ≤ 20px. Mitochondrial were identified using the “find spots” building block. The parameters used in the “find spots” block were as follows: method, B; detection sensitivity: 0.50 and splitting sensitivity: 0.50. Multiple parameters of neuron morphology -such as neurite roots, extremities, segments, branching nodes and length and mitochondrial morphology - mitochondrial area, perimeter, length, and width were measured.

#### Isolation of RNA, RNA-Seq and RT-qPCR

RNA was extracted using the RNeasy Mini Kit (50) (Catalog no: 74104, Qiagen) as per manufacturer’s manual. Briefly, cells were lysed using the RLT lysis buffer and homogenized by vertexing for 1 min. 1 volume of 70% ethanol was added to the homogenized sample. The sample was then transferred to a RNeasy spin column placed in a 2 mL collection tube. The samples were centrifuged for 15 s at 8000 x g and the flow through was discarded. Buffer RW1 was added to the RNeasy spin column and centrifuged for 15 s at 8000 x g and the flow through was discarded. Buffer RPE was added to the RNeasy spin column and centrifuged for 15 s at 8000 x g and the flow through was discarded. Then, buffer RPE was added to the RNeasy spin column and centrifuged for 2 min at 8000 x g. The 2 min spin should dry the spin column membrane. The RNeasy spin column was transferred to a new collection tube. RNAse-free water was added to the spin column membrane and the column was centrifuge for 1 min at 8000 x g to elute the RNA. Fluorometric Quantification was performed using the QubitTM 4 Flourometer (Catalog no: Q33239, Thermo Fisher Scientific) and purity was measured using the NanoDrop® ND-1000 UV–Vis Spectrophotometer.

RNA-seq data production and analysis was performed by Australian Genome Research Facility. The data is made publicly available on Gene expression Omnibus: accession number GSE233258.

RT-qPCR validation experiments were performed by Garvan Molecular Genetics, Sydney. The expression of genes GAD1 and GAD2 were analyzed using these primers: GAD1 forward CTTGTGAGTGCCTTCAAGGAG GAD1 reverse TGCTCCTCACCGTTCTTAGC GAD2 forward CTCGAAGGTGGCTCCAGTG GAD2 reverse CTCCCAAGGGTTGGTAGCTG.

#### Western blot analysis of paraplegin and synaptophysin expression

Neurons were harvested using accutase (Catalog no: A1110501, Stem cell technologies). Protein was extracted from the neuron cell pellet using a cell lysis buffer (Catalog no: C3228, Sigma) with 100X Halt Protease Inhibitor Cocktail (Catalog no: 78429, Thermo Fisher Scientific). Protein concentration was measured using the Pierce™ BCA Protein Assay Kit (Catalog no: 23225, Thermo Fisher Scientific) following manufacturers protocol. 10 μg of protein sample was resolved on a NuPAGE 4–12% BT gel (Catalog no: NP0335BOX, Thermo Fisher Scientific) with NuPAGE MOPS SDS running buffer (20X) and electro-transferred on to a polyvinylidene difluoride membrane using NuPAGE Transfer Buffer (20X). The membrane was blocked in 3% non-fat dry milk blocking buffer in Tris-buffered saline with 0.1% Tween® 20 detergent for 1 h at room temperature. The membrane was incubated overnight with primary antibodies diluted in 3% non-fat dry milk blocking buffer at 4°C: anti-*SPG7* (dilution 1: 1000, Catalog no: TA504424, Origene) and anti-Synaptophysin (dilution 1:700, Catalog no: ab32127, Abcam). Following this, incubation with secondary antibodies anti-mouse (Catalog no: STAR207P, Biorad) and anti-Rabbit (Catalog no:111–035-144, Jacson Immuno Research) was performed at room temperature for 1 h. This was followed by repeated washing with Tris-buffered saline with 0.1% Tween® 20 detergent. Immunoreactive bands were visualized using Chemiluminescent, SuperSignal™ West Femto Maximum Sensitivity Substrate (Catalog no: 34096, Thermofisher Scientific). The bands were imaged on the ImageQuant RT ECL imaging system (GE Healthcare). Band intensities were measured using Image Lab software (Bio-Rad). Paraplegin and synaptophysin intensities were normalized against GAPDH expression to obtain relative expression levels.

#### Electrophysiological recordings – whole cell patch clamping

We used whole-cell patch-clamp recordings to monitor neuron synaptic activity. Standard whole-cell patch-clamp techniques were used to study the functional maturation of the neurons. The patch clamp experiments were performed using the List EPC-7 patch-clamp amplifier (List, Darmstadt, Germany). Currents were low-pass-filtered, sampled, and digitized at 0.2 kHz with a PowerLab 4/30 data acquisition interface (AD Instruments, Sydney, NSW, Australia) attached to a Macintosh computer. Patch-clamp pipettes were manufactured from borosilicate tubes (Modulohm, Herley, Denmark).

Artificial cerebrospinal fluid (ACSF) was used as the bath solution. It composed of (in mM): 126 NaCl, 3 KCl, 1.2 NaH_2_PO_4_, 1.3 MgSO_4_, 2.4 CaCl_2_, 26 NaHCO_3_, 10 D-glucose (pH7.6, Osmo 300 mOsm/Kg). The cells were preincubated with ACSF at 37°C for 1 h before the start of the patch-clamp experiment. The electrode pipettes (7–10 MΩ) were filled with (in mM): 148 KCl, 2 MgCl_2_, 0.2 EGTA, 10 HEPES, 4 Na_2_ATP except the tips of the pipettes, which were filled with (in mM): 126 K-gluconate, 2 KCl, 2 MgCl_2_, 0.2 EGTA, 10 HEPES, 4 Na_2_ATP (pH7.3, Osmo 295 mOsm/Kg). A protocol was applied to deliver voltage pulses to step the membrane potential from −100 mV to +70 mV (in four steps from +40 mV to +70 mV with 10 mV intervals) and the current responses were recorded. All patch-clamp experiments were performed at room temperature.

## Results

### SPG7 patient neural progenitors with aberrant mitochondrial morphology and function develop to form mature neurons with reduced neurite complexity, length, viability and increased degeneration

**Figure 1:**
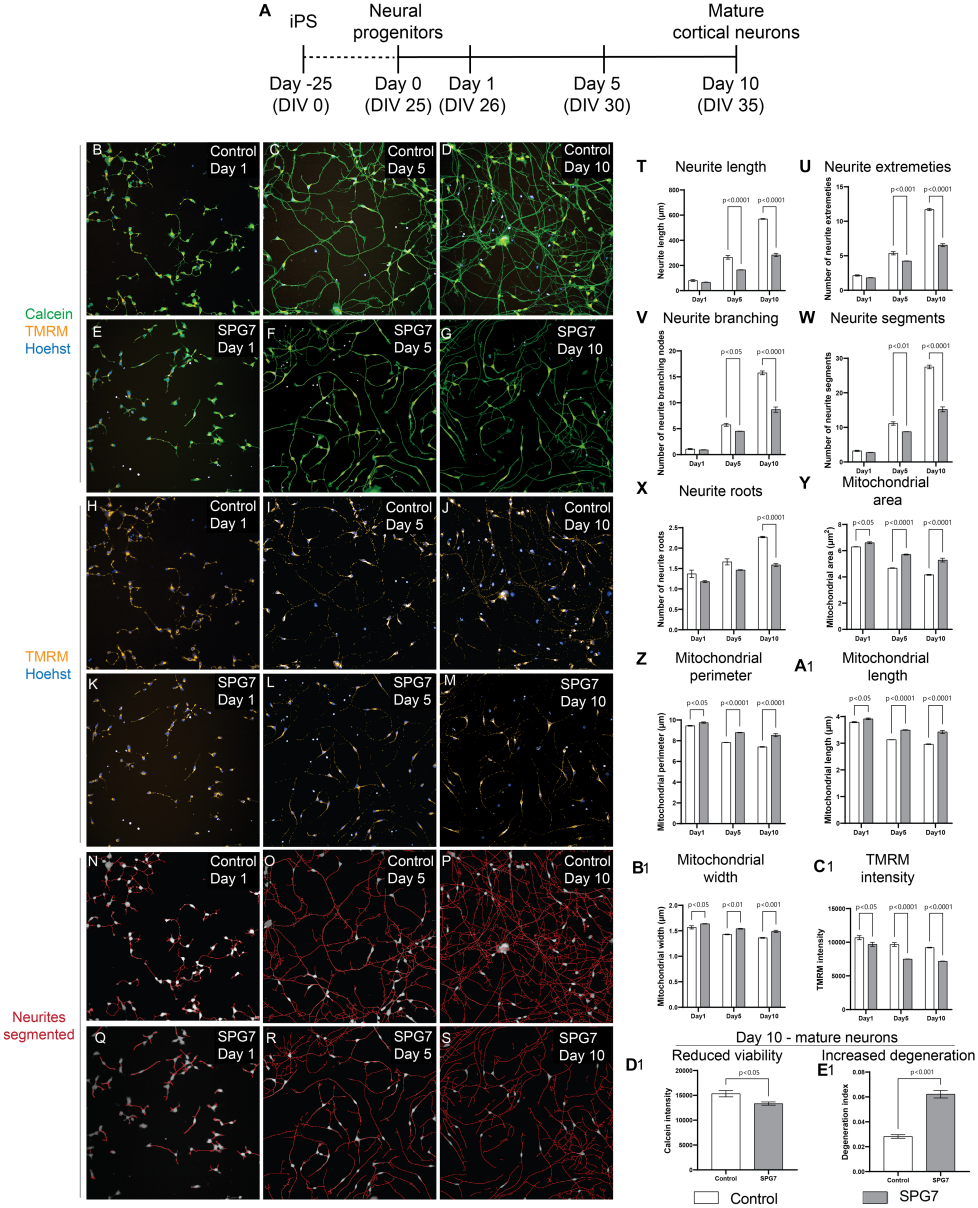
Neuronal and mitochondrial phenotypes in live HSP-SPG7 patient neural progenitors and mature cortical neurons. High throughput imaging and analysis was used to evaluate neuron and mitochondrial morphology. **(A)** Shows the timeline to generate neural progenitors and mature cortical neurons. **(B–G)** Images of live control and patient neural progenitors and mature cortical neurons labelled with calcein – to identify viable cells, TMRM – to identify mitochondrial and hoechst – to identify nucleus. **(H–M)** Images of the cells presented in **(B–G)** with with TMRM and Hoechst label without the calcein label. **(N–S)** Neurites were segmented and identified using automated image analysis in control and patient neural progenitors and mature cortical neurons. **(T–X)** Multiple parameters of neurite morphology were analyzed in control and patient neural progenitors and mature cortical neurons. These parameters include neurite length **(T)**, extremities **(U)**, branching **(V)**, segments **(W)** and roots **(X)**. **(Y–B1**) Multiple parameters of mitochondrial morphology and membrane potential were analyzed in control and patient neural progenitors and mature cortical neurons. These parameters include **(Y)** mitochondrial area, **(Z)** perimeter, **(A1)** length, **(B1)** width and TMRM intensity **(C1)**. **(D1,E1)** Cell viability **(D1)** and **(E1)** neurite degeneration in Day 10 mature control and patientneurons. Data is presented as Mean ± SEM. Scale bar: 100 μm.

To identify neuronal and mitochondrial related defects in HSP-*SPG7* patient-derived neurons, we measured and compared neuronal and mitochondrial morphological phenotypes and mitochondrial membrane potential between patient cells and healthy control cells at multiple timepoints (Figure 1A) as they differentiated from neural progenitors to mature neurons.

Live patient and control cells were labelled with calcein (green) - to identify viable cells, Tetramethylrhodamine, methyl ester (TMRM) (red) – to identify healthy mitochondrial and hoechst (blue) – to identify nucleus (Figures 1B–M). The cells were imaged and analyzed using an automated high throughout imaging and analysis microscope (PhenixPlus, Perkin Elmer). For image analysis, the neurites were first segmented and identified (Figures 1N–S). Then, multiple parameters indicative of neurite complexity such as neurite roots, branching, extremities, segments, and length were measured (Figures 1T–X). For all measurements of neurite parameters, Two-Way ANOVA test indicated a significant effect of disease status (*p* < 0.0001) and neurite development as they progressed from neural progenitors to form mature neurons (*p* < 0.0001). Šídák’s post-hoc multiple comparisons test indicates that the neurite complexity and length measurements in patient cells is comparable to control cells at Day 1, i.e., the neural progenitor phase. But the neurite complexity and length measurements in patient cells are significantly lower compared to control cells at Days 5 and 10 as they develop to form mature neurons (Figures 1T–X).

To identify mitochondrial morphological and functional abnormalities, we measured multiple parameters of mitochondrial size such as mitochondrial area, perimeter, length, and width and membrane potential (Figures 1Y–C1). For all measurements of mitochondrial morphology parameters and membrane potential, Two-Way ANOVA test indicated a significant effect of disease status (*p* < 0.0001) and neurite development as they progressed from neural progenitors to form mature neurons (*p* < 0.0001). Šídák’s post-hoc multiple comparisons test indicates that the mitochondrial size related parameters in patient cells are significantly higher compared to control cells at Day 1 during the neural progenitor phase and at Days 5 and 10 as they develop to form mature neurons (Figures 1Y–B1). Šídák’s post-hoc multiple comparisons test indicates that the mitochondrial membrane potential in patient cells is significantly lower compared to control cells at Day 1 during the neural progenitor phase and at Days 5 and 10 as they develop to form mature neurons (Figure 1C1).

In summary, the defect of reduced neurite complexity and length was absent at Day 1, i.e., the neural progenitor phase but was present at Day 5 and Day 10, i.e., mature neurons. However, the abnormalities in mitochondrial size and membrane potential were seen at all three time-points, i.e., at Day 1 the neural progenitor phase, and Day 5 and Day 10, i.e., in mature neurons. This shows that mitochondrial defects developed before neuronal defects.

Evaluation of neuronal viability and degeneration in mature cortical neurons at Day 10 showed that compared to control neurites, patient neurites had reduced viability (Figure 1D1) and increased neurite degeneration (Figure 1E1).

### *SPG7* patient neurons have less complex and shorter neurites and increased degeneration but they express mature cortical neuron markers

**Figure 2:**
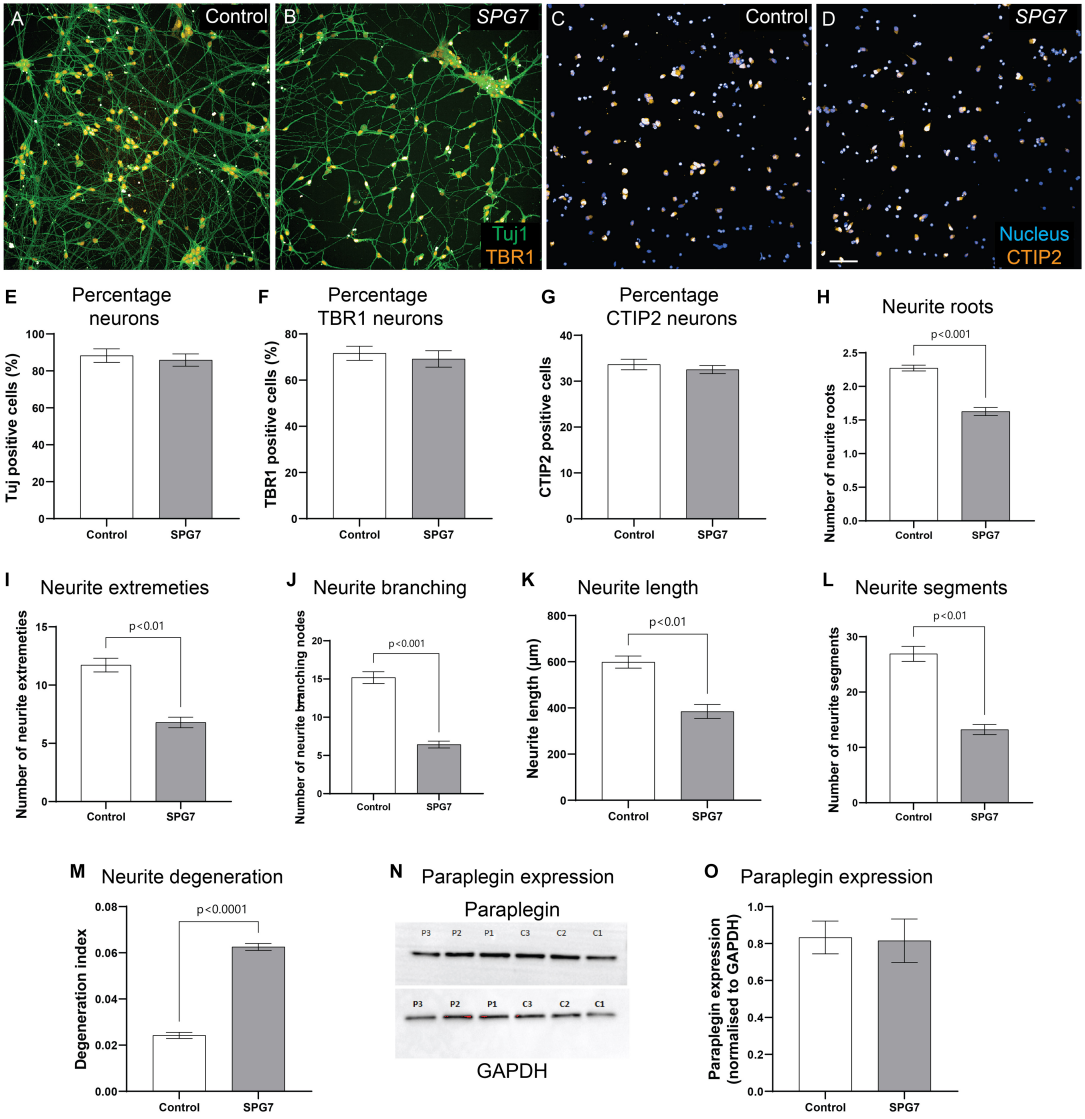
Neuronal phenotypes confirmed in fixed and immunolabelled HSP-SPG7 patient neurons. **(A–D)** Control and patient mature cortical neurons express Tuj1 – neuronal marker and mature cortical neuronal markers - TBR1 **(A,B)** and CTIP2 **(C,D)**. **(E–G)** The proportion of Tuj1, TBR1 and CTIP2 positive cells are comparable between controls and patients. **(H–L)** Multiple parameters of neurite morphology were analyzed in control and patient mature cortical neurons. These parameters include neurite **(H)** roots, **(I)** extremities, **(J)** branching, **(K)** length and **(L)** segments. **(M)** Axonal degeneration of control and patient neurites in mature cortical neurons. **(N,O)** Paraplegin expression was measured in control and patient mature cortical neurons. Data is presented as Mean ± SEM. Scale bar: 100 μm.

In Figure 1, neurite morphology was evaluated in cells labelled with calcein – a viability dye. Calcein is not neuron specific. To confirm the neuronal phenotype defect in mature cortical neurons, we fixed and immunolabelled the neurons with Tuj1 – a neuronal marker and mature cortical neuronal markers TBR1 and CTIP2 (Figures 2A–D). The expression of Tuj1, TBR1 and CTIP2 markers were comparable between the patient and control neurons (Figures 2E–G).

Similar to our findings in calcein labelled neurites (Figure 1), Tuj1 labelled neurites showed that compared to control neurons, patient neurons had reduced neurite roots (Figure 2H), extremities (Figure 2I), branching nodes (Figure 2J), length (Figure 2K), segments (Figure 2L) and increased neurite degeneration (Figure 2M). In summary, although HSP-*SPG7* patient neurons were less complex and shorter and had increased degeneration they expressed mature cortical neuron markers.

*SPG7* encodes for paraplegin protein. To test if paraplegin expression is altered in patient neurons, we measured paraplegin expression in patient and control neurons (Figure 2N). Paraplegin expression was comparable between patient and control neurons (Figure 2O). We have previously observed this effect in HSP-*SPG7* patient derived olfactory neurosphere-derived cells (Wali et al., 2020). It is plausible that the paraplegin protein expressed is non-functional.

### *SPG7* patient neurons have downregulated synapse related gene expression, reduced synaptic activity and increased oxidative stress gene expression

**Figure 3:**
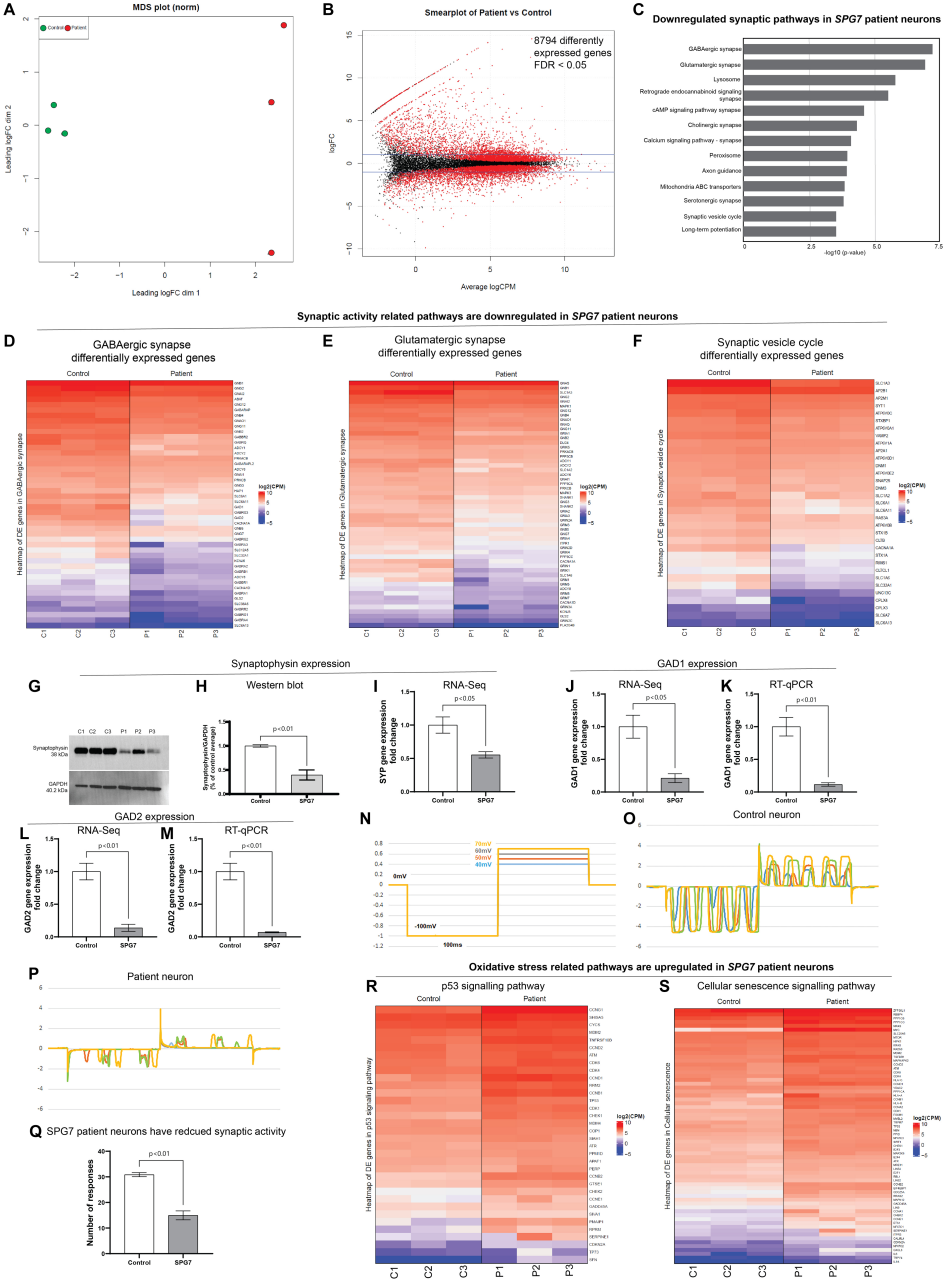
RNA-Seq analysis of HSP-SPG7 patient neurons. RNA-Seq analysis was performed using patient and healthy control neurons to identify gene expression pathways affected in *HSP-SPG7* patient neurons. **(A)** Multidimensional scaling analysis was used to visualize the level of similarity in the gene expression of the patient and control neurons. **(B)** Smearplot shows a large number of differently expressed genes. **(C)** Pathway enrichment analysis was performed to understand which pathways/gene networks the differentially expressed genes are implicated in. Synaptic pathways was FIGURE 3 (Continued)downregulated in patient neurons. **(D–F)** List of genes that expressed lower in patient neurons compared to control neurons in the GABAergic synapse **(D)**, Glutamergic synapse **(E)** and synaptic vesicle cycle pathway **(F)**. **(G,H)** Western blot analysis and **(I)** RNA-Seq consistently showed reduced expression of synaptophysin expression. **(J–M)** RT-qPCR validated the findings of RNA-Seq based GAD1 and GAD2 gene expression. **(N–Q)** Whole cell patch clamping was used to measure neuronal synaptic activity in control and patient neurons. **(R,S)** List of genes that expressed higher in patient neurons compared to control neurons in the oxidative stress related p53 **(R)** and cellular senescence **(S)**.

To evaluate the gene expression pathways affected in HSP-*SPG7* patient neurons, we performed RNA-Seq analysis on HSP-*SPG7* patient and healthy control neurons. For this we performed gene expression quantification followed by differential expression analysis of genes and pathway enrichment analysis. First, multidimensional scaling analysis was used to visualize the level of similarity in the gene expression of the patient and control neurons. The multidimensional scaling plot illustrated a clear distinction in gene expression profiles between patient and control neurons (Figure 3A).

Second, the differential gene expression analysis of patient and control neurons identified 8,794 significant differentially expressed genes - those with False discovery rate < 0.05 (Figure 3B). Third, pathway enrichment analysis was performed to understand which pathways/gene networks the differentially expressed genes are implicated in. This analysis showed that the genes related to synaptic function were majorly downregulated in patient neurons (Figure 3C). These pathways included GABAergic and Glutamergic synapse, neurotransmitter signaling, axon guidance and synaptic vesicle cycle. Figures 3D–F shows the genes differentially expressed in the GABAergic synapse, Glutamergic synapse and synaptic vesicle cycle pathways. All the genes listed in these pathways (Figures 3D–F) are expressed significantly lower (reported value of *p* <0.05) in patient neurons compared to control neurons. The colour code in the heatmaps refer to the levels of expression of genes following a log2 (Counts Per Million) transformation where the genes are ordered from highest expression levels to lowest. This means that the first gene on the list has much higher levels of expression compared to the second gene, so on and so forth, but all genes presented have reduced expression in patient neurons compared to control neurons. Note that the true differences in expression levels for the most highly expressed genes may be obfuscated due to the log scale.

To validate RNA-Seq findings, we performed western blotting and RT-qPCR for selected genes. For example, western blotting (Figures 3G,H) confirmed that the RNA-Seq finding that the expression levels of synaptophysin was lower in patient neurons compared to control neurons (Figure 3I). RT-qPCR evaluation of expression of genes GAD1 and GAD2 validated RNA-Seq findings that the GAD1 and GAD2 gene expression levels was lower in patient neurons compared to control neurons (Figures 3J–M).

In summary, the RNA-Seq results showed that the genes related to synaptic pathways were reduced in patient neurons compared to control neurons. To test the functional relevance of this finding, we performed whole cell patch clamping (Figures 3N–Q). The neurons in both groups showed inward and outward currents in response to the four steps of voltage pulses. However, the number of the current responses produced by the control neurons were significantly greater than those produced by the patient neurons (Figures 3N–Q). This result confirmed that the gene expression and function of synapse was reduced in patient neurons.

Dysfunctional mitochondrial can impair synaptic activity and contribute to oxidative stress leading to DNA damage and apoptosis (Cai and Tammineni, 2017). Further evaluation of the RNA-Seq data showed that gene expression pathways related to oxidative stress, i.e., p53 signaling (Figure 3R) and cellular senescence (Figure 3S) are upregulated in patient neurons compared to control neurons, indicating that the patient neurons are under oxidative stress.

### Pharmacological rescue of neurite and mitochondrial defects in *SPG7* neurons

**Figure 4:**
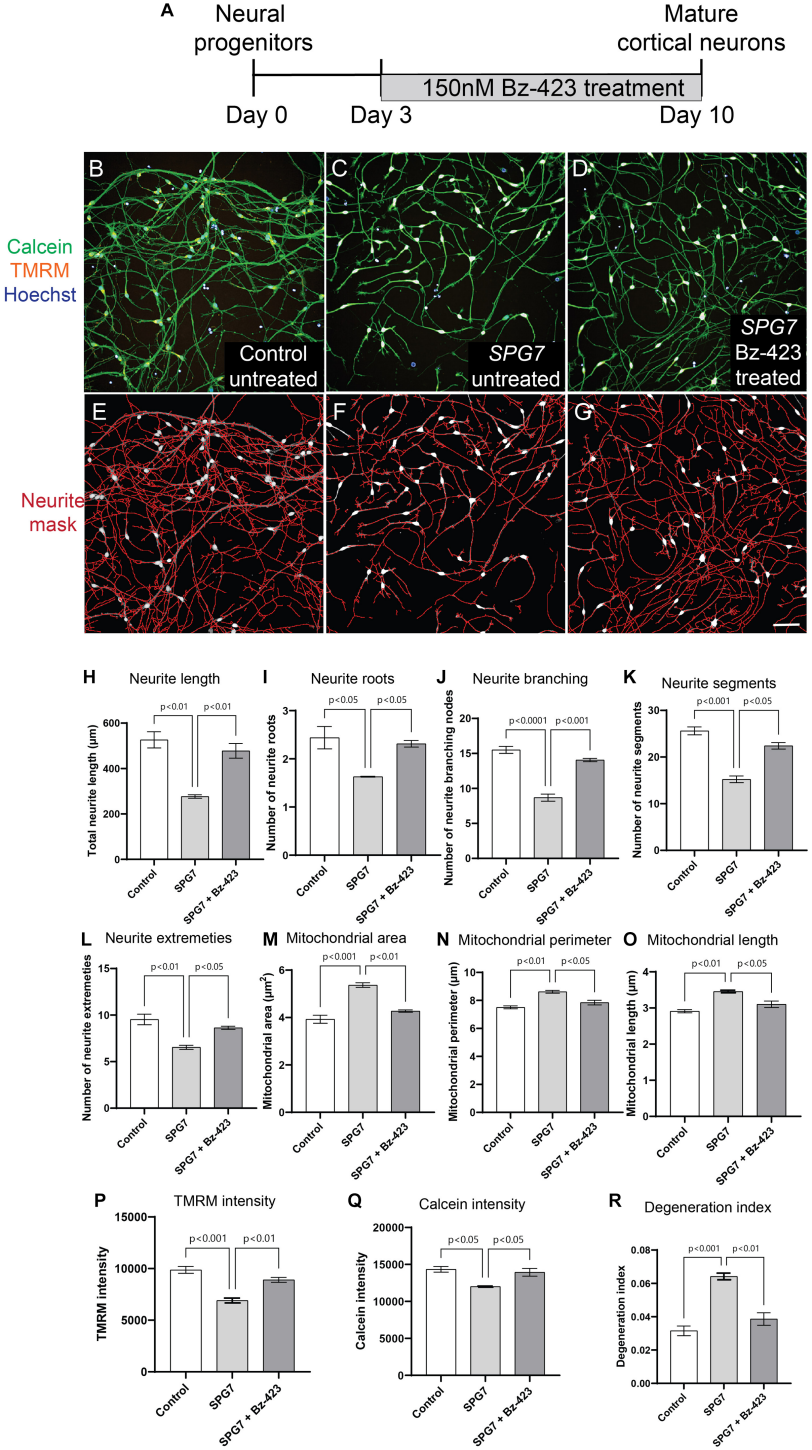
Pharmacological rescue of neuronal and mitochondrial phenotypes in HSP-SPG7 patient neurons. **(A)** Shows the Bz-423 drug treatment timeline. **(B–D)** Live untreated control, untreated patient and Bz-423 treated patient neurons labelled with calcein – to identify cells, TMRM – to identify mitochondrial and hoechst – to identify nucleus. **(E–G)** Neurites were segmented and identified using automated image analysis in untreated control, FIGURE 4 (Continued)untreated patient and Bz-423 treated patient neurons. **(H–L)** Multiple parameters of neurite morphology were analyzed in untreated control, untreated patient and Bz-423 treated patient neurons. These parameters included neurite **(H)** length, **(I)** roots, **(J)** branching, **(K)** segments and **(L)** extremities. **(M–O)** Multiple parameters of mitochondrial morphology and membrane potential were analyzed in untreated control, untreated patient and Bz-423 treated patient neurons. These parameters included mitochondrial **(M)** area, **(N)** perimeter and **(O)** length and **(P)** TMRM intensity. **(Q–R)** Viability and axonal degeneration of untreated control, untreated patient and Bz-423 treated patient neurons. Data is presented as Mean ± SEM. Scale bar: 100 μm.

To test if the neurite defects – reduced complexity, reduced viability and increased degeneration seen in HSP-*SPG7* patient neurons are a consequence of mitochondrial dysfunction, we treated patient neurons with Bz-423, a drug shown to be effective in rescuing mitochondrial function and neurological gait impairment in a *SPG7* mice model (Sambri et al., 2020). Patient neurons were treated for 7 days, with Bz-423 treatment initiated 3 days after seeding neural progenitors (Figure 4A). As described in Figure 1, patient (untreated and treated) and control neurons were labelled with calcein, TMRM and hoechst. The neurons were imaged, and multiple parameters indicative of neurite complexity and length and degeneration and mitochondrial size and function were measured (Figures 4B–G).

Bz-423 treatment restored neurite and mitochondrial defects in patient neurons back to control neuron levels (Figures 4H–R). ANOVA indicated a significant effect of treatment (*p* < 0.001). Tukey’s post-hoc multiple comparisons indicate that all neurite complexity, length (Figures 4H–L), mitochondrial morphology and membrane potential (Figures 4M–P), neuronal viability (Figure 4Q) and neurite degeneration (Figure 4R) in untreated patient neurons were significantly different from untreated control neurons and patient neurons treated with Bz-423.

## Discussion

We have established a HSP*-SPG7* patient neuronal cell model using patient-derived iPS cell differentiated neural progenitor cells and mature cortical neurons. Our results show that compared to control neural progenitor cells, patient neural progenitor cells have aberrant mitochondrial morphology with increased mitochondrial size and dysfunctional mitochondrial with reduced mitochondrial membrane potential. Patient neural progenitor cells developed to form mature cortical neurons with multiple mitochondrial and neuronal defects – increased mitochondrial size, reduced membrane potential, reduced neurite complexity and length, reduced synaptic gene and protein expression, reduced viability and increased axonal degeneration. Treatment of patient neurons with Bz-423, a mitochondria permeability pore regulator, restored the mitochondrial and neurite morphological defects and mitochondrial membrane potential back to control neuron levels and rescued the low viability and increased degeneration in patient neurons. We used Bz-423 at low nano molar concentrations (150 nM) known to be safe and effective in rescuing mitochondrial defects (Sambri et al., 2020). This study establishes a direct link between mitochondrial and neuronal defects in HSP*-SPG7* patient neurons. We present a strategy for testing mitochondria targeting drugs to rescue neuronal defects in HSP*-SPG7* patient neurons.

Recent studies have highlighted the possibility of dysregulated mPTP to be the leading cause of mitochondrial dysfunction in HSP*-SPG7*. To test if the neurite defects seen in patient neurons are a consequence of mitochondrial dysfunction, we treated patient neurons with Bz-423, a mPTP modulating drug that has shown to be effective in rescuing mitochondrial function and neurological gait impairment in HSP*-SPG7* mice model (Sambri et al., 2020). mPTP regulates the mitochondrial permeability transition, which refers to a sudden increase in the inner mitochondrial membrane permeability. mPTP can exist in low and high conductance modes (Zoratti and Szabò, 1995). mPTP is normally in its low conductance mode, where it permits the diffusion of ions below 300 Da such as K+ and Ca2+. Under pathological conditions such as increased mitochondrial matrix calcium accumulation or increased oxidative stress, mPTP is in its high conductance state. In its high conductance state mPTP permits free unrestricted diffusion of large molecules up to 1.5 kDa across the inner mitochondrial membrane and results in the mitochondrial matrix swelling (Kwong and Molkentin, 2015). One major consequence of mPTP high conductance state is that the inner mitochondrial membrane can no longer maintain a barrier to protons which leads to dissipation of the proton motive force, resulting in uncoupling of oxidative phosphorylation and dissipation of the mitochondrial membrane potential. Thus, preventing mitochondria from making ATP. HSP*-SPG7* patient cells have increased oxidative stress (Wali et al., 2020). This increased oxidative stress can lead to mPTP is in its high conductance state. To test if this effect is relevant to HSP*-SPG7* patient neurons, we measured multiple parameters of mitochondrial size – length, width, area, and perimeter and mitochondrial membrane potential. Prolonged opening of the mPTP leads to increased mitochondrial size. Our evaluation showed increased mitochondrial size and reduced membrane potential in patient neural progenitor cells and mature neurons. The patient vs. control difference was amplified in mature neurons. Consistent with this, a 6-month-old paraplegin deficient mice showed the presence of swollen mitochondria in spinal cord axons (Sambri et al., 2020). Treatment of HSP*-SPG7* patient neurons with low nano molar concentrations of mPTP targeting drug Bz-423, shown to rescue the defect of swollen mitochondrial and motor impairment in paraplegin-deficient HSP-*SPG7* mice model (Giorgio et al., 2013; Sambri et al., 2020), also restored the defect of increased mitochondrial size in our patient neurons back to control neuron levels, further indicating that the increased mitochondrial size and reduced membrane potential in HSP*-SPG7* patient neurons was a consequence of mPTP dysfunction.

Despite weighing only 2% of the human body weight, the adult brain consumes about 20% of all energy generated (Attwell and Laughlin, 2001). Healthy mitochondrial is essential in maintaining synaptic activity. To maintain synaptic activity, mitochondrial is generated in the cell soma and transported to dendrites where they are distributed around the synapse to actively generate energy required for synaptic activity (du et al., 2010). Along with meeting energy demands, the mitochondrial is involved in (a) maintaining ion gradients across the cellular membrane for axonal and synaptic membrane potentials (Attwell and Laughlin, 2001), (b) mobilizing synaptic vesicles to release sites (Verstreken et al., 2005) and (c) supporting synaptic vesicle release (Sun et al., 2013). Dysfunctional mitochondrial has shown to cause impaired synaptic activity in multiple neurodegenerative diseases (du et al., 2010; Cai and Tammineni, 2017). Dysfunctional mitochondrial also release reactive oxygen species causing oxidative stress, which can result in DNA damage, and apoptosis. Consistent with this, our *SPG7* patient neurons have dysfunctional mitochondrial, reduced synaptic gene expression and function, upregulated oxidative stress pathways, reduced viability, and increased degeneration.

Impaired neurite complexity and length has been described in many other forms of HSP including *SPG4* (Rehbach et al., 2019), *SPG11* (Pérez-Brangulí et al., 2014), *SPG15* (Denton et al., 2018) and *SPG48* (Denton et al., 2018). *SPG15* and *SPG48* HSP patient-derived iPS telencephalic glutamatergic and midbrain dopaminergic neurons had reduced neurite number, length, and branching, altered mitochondria morphology with reduced mitochondrial length and density and dysfunctional mitochondria with reduced mitochondrial membrane potential. Treatment of patient neurons for 48 h with an inhibitor of mitochondrial fission rescued the mitochondrial and neurite deficits (Denton et al., 2018).

Our results showed that treating patient neurons in their development phase can avert disease-associated mitochondrial and neuronal phenotypes including neuronal degeneration in mature neurons. This approach opens up the possibility of not just reversing the damaged neurons in adult patients but possibly preventing the development of disease-associated neuronal phenotypes if treatment can be initiated in the early stage of disease onset. It is well accepted that the degeneration of neuronal cells occurs about a decade before the clinical symptoms begin. In this scenario, early disease diagnosis and treatment can be key in reducing the severity of the disease.

Unfortunately, as mentioned in the introduction, at higher concentrations Bz-423 is anti-proliferative and cytotoxic, making it challenging to translate this to the clinical. In this study, we use Bz-423 for its ability to rescue mitochondrial defects in neurons at low nano molar concentrations. In the future, using the (a) assays described in this manuscript, (b) understanding of mitochondrial and neuronal defects in HSP-*SPG7* patient neurons and (c) the drug treatment approach described here, we will screen for FDA approved drugs to repurpose them for HSP-*SPG7*.

Our assays evaluating neuron and mitochondrial presented here has multiple advantages for identifying disease-associated effects that can be used as cellular biomarkers to identify new potential drug treatment candidates: (1) measures multiple parameters of neurite complexity including neurite length, roots, branching, segments and extremities and degeneration (2) measures multiple parameters of mitochondrial morphology and function (3) the assay is high throughput and performed in 96 well plates allowing testing large number of cells across multiple control and patient cell lines and treatment effects in a single experiment avoiding sample to sample and batch to batch variability (4) automated imaging can image large number of cells in a relatively short amount of time (100,000 cells in 1 h) (5) automated image analysis pipeline can analyse all cells using the same image analysis parameters without any user bias and (6) The cell permeable dyes used in this assay, i.e., Calcein, TMRM and Hoechst are at least 10x cheaper than antibodies. This is ideal for drug testing and screening assays that involves testing drug treatment effectiveness and cytotoxicity in a large number of drugs at multiple different concentrations.

## Data availability statement

The datasets presented in this study can be found in online repositories. The names of the repository/repositories and accession number(s) can be found at: https://www.ncbi.nlm.nih.gov/geo/, GSE233258.

## Ethics statement

The studies involving humans were approved by Human Research Ethics Committee at Northern Sydney Local Health District Human Research Ethics Committee, Australia (2019/ETH08193) and written informed consent was obtained from all participants. The studies were conducted in accordance with the local legislation and institutional requirements. The participants provided their written informed consent to participate in this study.

## Author contributions

GW and CS designed the study and provided funding. CS and KK recruited patients to the study. YL, EL, and GW performed the experiments. GW performed data analysis and wrote the first draft. MD analyzed the whole cell patch clamp data. All authors contributed to the article and approved the submitted version.

## Conflict of interest

The authors declare that the research was conducted in the absence of any commercial or financial relationships that could be construed as a potential conflict of interest.

## Publisher’s note

All claims expressed in this article are solely those of the authors and do not necessarily represent those of their affiliated organizations, or those of the publisher, the editors and the reviewers. Any product that may be evaluated in this article, or claim that may be made by its manufacturer, is not guaranteed or endorsed by the publisher.

## Supplementary material

The Supplementary material for this article can be found online at: https://www.frontiersin.org/articles/10.3389/fnins.2023.1231584/full#supplementary-material
