# Supplementary material for: Pharmacological rescue of mitochondrial and neuronal defects in SPG7 hereditary spastic paraplegia patient neurons using high throughput assays
Source: Front Neurosci. 2023 Sep 12;17:1231584. doi: 10.3389/fnins.2023.1231584 (PMC10520970; doi:10.3389/fnins.2023.1231584)
Supplement: Supplementary file 1 [file Data_Sheet_1.docx]

***Supplementary methods section: Characterization of iPS cell lines***

***Trilineage differentiation of iPS cells to test pluripotency*** To test the differentiation ability of the iPS cells, we differentiated iPS cells to the three germ layers i.e., endoderm, ectoderm and mesoderm using the STEMdiff^TM^ Trilineage differentiation Kit (Catalog no: #05230, Stem cell technologies) as per manufacturer manual. The kit includes lineage specific medias for directed differentiation to each germ layer within 7 days: ectoderm (7 days), mesoderm (5 days) and endoderm (5 days). Briefly, on day 0, 6-well plates were coated with Corning® Matrigel® hESC-Qualified Matrix (Catalog no: 354277, Corning). iPS cells were dissociated as single cells using the Gentle Cell Dissociation Reagent (Catalog # 100-0485, Stemcell dissociation reagent) and seeded at these densities for ectoderm (200,000 cells/cm^2^), endoderm (200,000 cells/cm^2^) and mesoderm (50,000 cells/cm^2^) differentiation. While seeding iPS cells as single cells, the cells were maintained in mTeSR™1 media with ROCK inhibitor Y-27632. From day 1 to day 5 or 7, the appropriate STEMdiff™ Trilineage medium was added to each well. The differentiation was assessed using germ line specific markers by flow cytometer.

***Flowcytometry based analysis of pluripotency and trilineage differentiation markers*** For flow cytometry-based analysis, both iPS cells and the trilineage differentiated cells were harvested as single cells using the using the Gentle Cell Dissociation Reagent (Catalog no: 100-0485, Stemcell dissociation reagent,). The cells were immunostained with primary and secondary antibodies using the Cytofix/Cytoperm Fixation/Permeabilization solution kit (Catalog no: 554714, BD Biosciences), as described before (Wali et al., 2021). The cells were fixed using the CytoFix reagent for 25 min. Cells were washed twice using the CytoPerm reagent. The cells were permeabilised and blocked using the CytoPerm reagent for 30min. The cells were incubated with primary antibodies for 1 h: anti-OCT4 (1:1000, Catalog no: ab19857, Abcam), anti-Sox2 (1:200, Catalog no: ab93689, Abcam), negative isotype control (1:1000, Catalog no: ab172730, Abcam), anti-Nestin (1:100, Catalog no: ab22035, Abcam), anti-Brachyury (1:100, Catalog no: ab20680, Abcam) or anti-GATA4 (1:100, Catalog no: ab84593, Abcam). Cells were washed twice using the CytoPerm reagent. The cells were incubated with appropriate anti-mouse or anti-rabbit secondary antibodies (1:500, A-11012 or A11028, Abcam) for 30 min. Cells were washed twice and resuspended in CytoPerm reagent. The samples were analysed on the BD LSR Fortessa flow cytometer and quantified using the BD FACS Diva™ software.

***Sanger sequencing to confirm patient mutation*** Mutations in *SPG7* patient-derived iPS cells were confirmed by sanger sequencing. DNA was isolated from the iPS cells using the DNeasy Blood & Tissue kit following manufacturer’s guidelines. Briefly, 180µl of lysis buffer ALT () and 20 µl of proteinase K were added to the cell pellet. The sample was incubated at70^O^C for 10 min. Then, 200µl of absolute ethanol was added and vortexed for 15 seconds. The sample was then added onto the QIAamp mini spin column. The sample was centrifuged at 6000 x g for 1 minute. The residue was discarded. Then, 500 µl of AW1 and AW2 buffers were added consequently, followed by centrifuging at 20,00 x g (14, 000 rpm) for 3 minutes. The QIAamp mini spin column was dried by centrifuging at maximum speed for 1 minute. The DNA was collected by adding buffer AE and running the elute through the column 3 times consequently.

Primer design and sanger sequencing was performed by Garvan Molecular Services, Sydney. Here are the primers used for the patient samples. Patient 1: Primer1, SS832_GW_1529F, tgtaaaacgacggccagtCTAGGCTTGAGCCCGACTGTC; Primer2, SS833_GW_1529R, caggaaacagctatgaccCACAGAGGAGGCCTCGATGC; Primer3, SS834_GW_1449F, tgtaaaacgacggccagtGTACCACAGACCATGTCATCG; Primer4, SS835_GW_1449R, caggaaacagctatgaccCAGTGAGTGCAGCCGGTGGG. Patient2: Primer1, SS828_GW_415F, tgtaaaacgacggccagtGAGCTTTCCTGAGGAAGCTC; Primer2, SS829_GW_415R, caggaaacagctatgaccCGCGCTGCACCTCGCCCTTG; Primer3, SS830_GW_941F, tgtaaaacgacggccagtCTGACTTCGCCCAGCTCCTTG; Primer4, SS831_GW_941R, caggaaacagctatgaccGGTAGAGGTCACCGCCAACC. Patient3: Primer1: CDSS155_SPG7_Int16_F, tgtaaaacgacggccagtGTGGCCTGTCCTGGGTGTCC; Primer2: CDSS156_SPG7_Ex17_R, caggaaacagctatgaccCTCTGGGCGTCGATCCACCTC; Primer3: SS832_GW_1529F, tgtaaaacgacggccagtCTAGGCTTGAGCCCGACTGTC; Primer4: SS833_GW_1529R, caggaaacagctatgaccCACAGAGGAGGCCTCGATGC.

***Cytology to evaluate chromosomal integrity of iPS cell lines*** A routine Global Screening Array (Australian Genome Research facility) was performed to check for copy number variants or allelic changes. Data was analysed using Genome Studio 2.0.

***Supplementary figures***

***Supplementary figure 1***


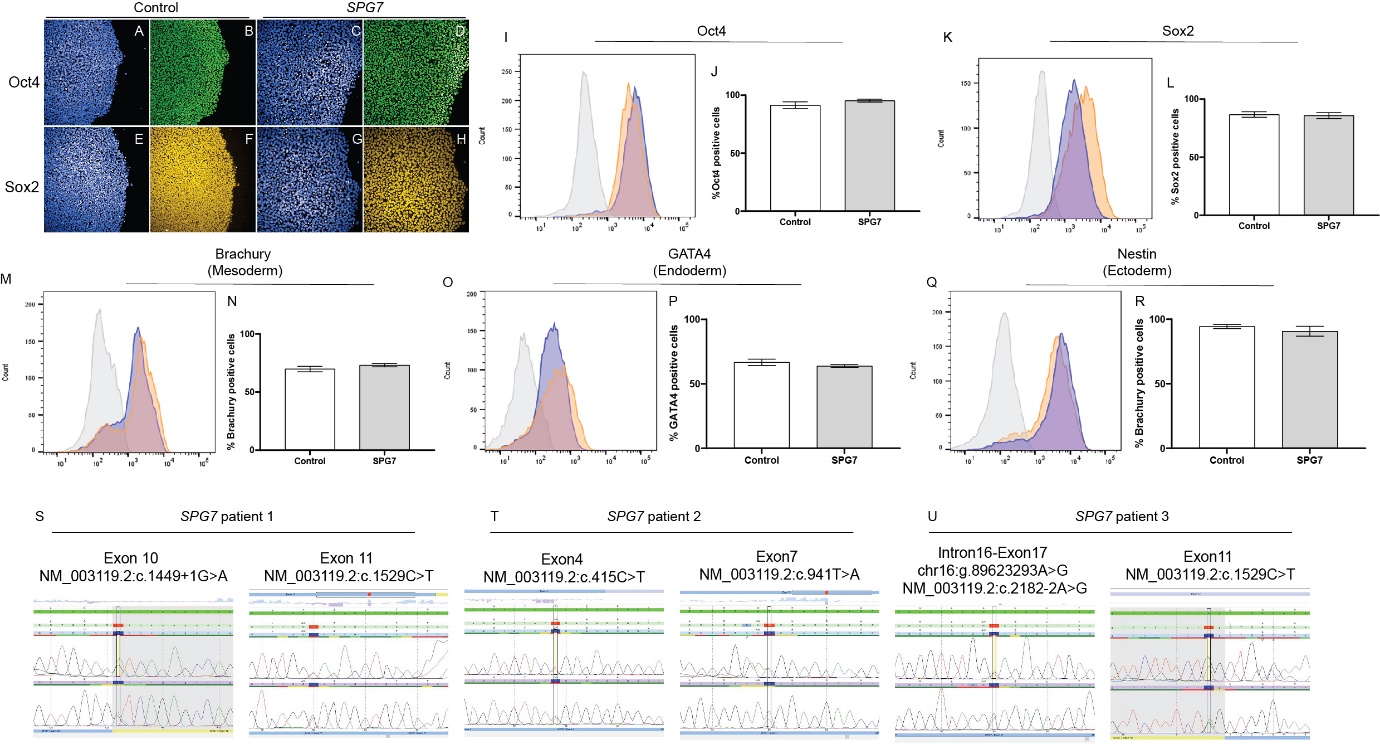


***Supplementary figure 1: Characterization of SPG7 patient and control iPS cells.*** (**A-H**) Control and patient iPS cells labelled with pluripotency markers Oct4 (green), Sox2 (yellow) and nuclei marker, Hoechst (blue). (**I-L**) Flow cytometry-based evaluation showed that the expression of Oct4 and Sox2 were comparable between the patient and control iPS cell lines. (**M-Q**) The differentiation ability of iPS cells to the three germ layers i.e., (**M**) mesoderm, (**O**) endoderm and (**Q**) ectoderm were comparable between control and patient iPS cell lines. (**S-U**) Sanger sequencing confirmed that the reprogrammed patient iPS cell lines were still carrying the disease mutations.

***Supplementary figure 2***


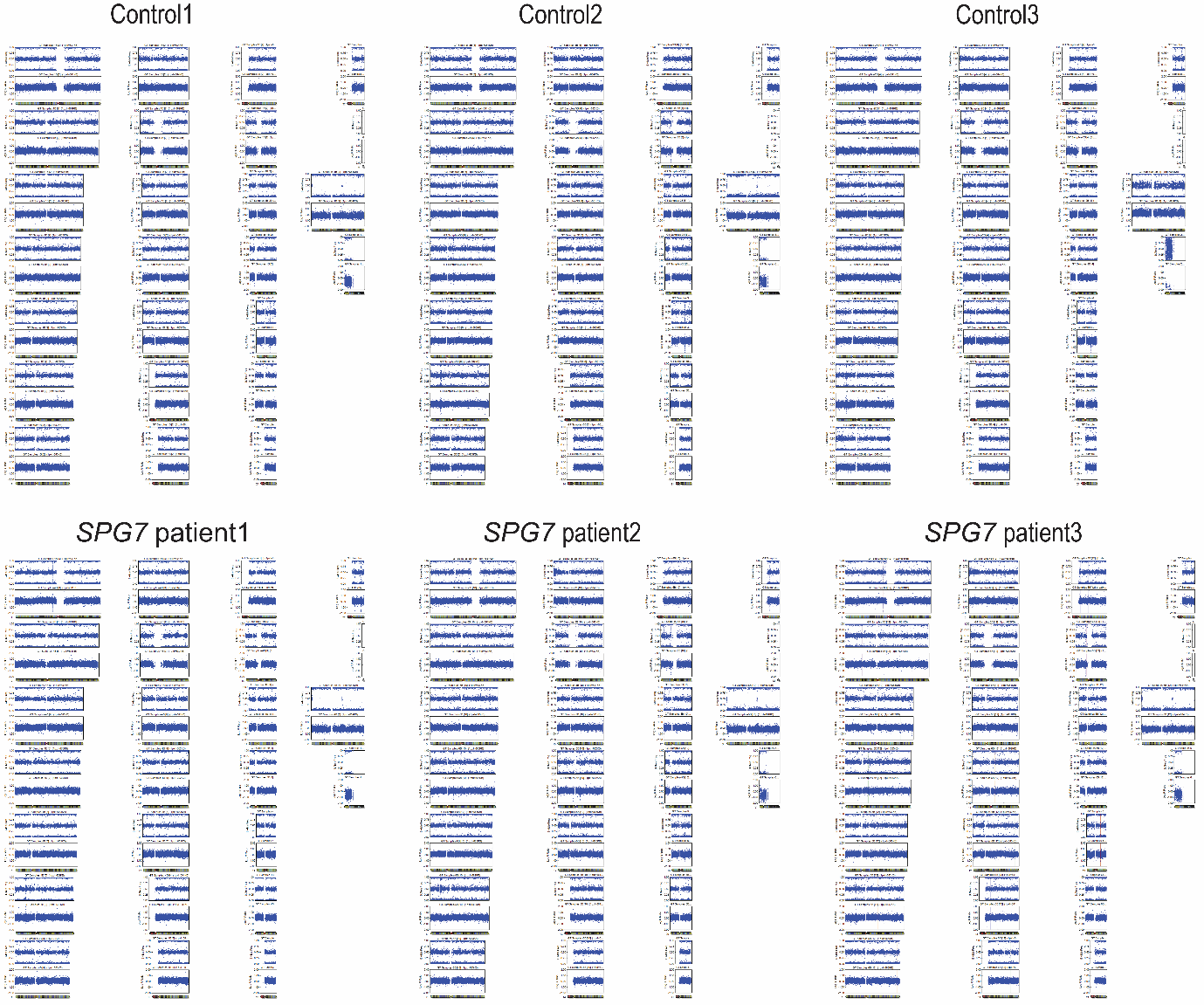


***Supplementary figure 2:*** No copy number variants or allelic changes were detected in patient or control iPS lines.
