# Supplementary material for: Pharmacological rescue of mitochondrial and neuronal defects in SPG7 hereditary spastic paraplegia patient neurons using high throughput assays
Source: Front Neurosci. 2023 Sep 12;17:1231584. doi: 10.3389/fnins.2023.1231584 (PMC10520970; doi:10.3389/fnins.2023.1231584)
Supplement: Supplementary file 2 [file Data_Sheet_2.docx]

Supplementary table1: Clinical features and mutation details of HSP patients with compound heterozygous disease-causing variants in *SPG7*

| **Study participant ID**  **Gene**  **Mutation(s)** | **Patient2**  NM_003119.4:c.941T>A  (p.Met314Lys)  NM_003119.4:c.415C>T  (p.Arg139Ter) | **Patient3**  NM_003119.4:c.2182-2A>G  NM_003119.4:c.1529C>T(p.Ala510Val) |
| --- | --- | --- |
| Patient ID | 18/027 | 18/273 |
| Ethnic background | European | European |
| Phenotype | Complex | Complex |
| Family History | AR | AR/Pseudodominant |
| Age at biopsy (years) | 51 | 47 |
| Age at onset (year) | 43 | 45 |
| UL amyotrophy | No | No |
| UL spasticity | No | Yes |
| UL weakness | No | Mild |
| UL reflexes | ++ | ++ |
| LL amyotrophy | No | No |
| LL spasticity | Severe | Moderate |
| LL weakness | No | No |
| LL reflexes | +++ | ++++ |
| Babinski | Yes | Yes |
| Sensory disturbance | No | N/A |
| Urinary disturbance | Yes | Yes |
| Ophthalmoplegia | Yes, complex ophthalmoplegia with impairment of upgaze. | Yes, complex |
| Ptosis | No | Yes, asymmetric ptosis – left eye worse than right |
| Dysarthria | Mild | No |
| Cerebellar ataxia | Yes | Yes |
| Walking aids | Wheelchair | No |
| Brain MRI | N/A | N/A |
| Spinal MRI | N/A | N/A |
| NCS/EMG | N/A | N/A |
| MEP | N/A | N/A |
| Muscle biopsy features of mitochondrial cytopathy | Not performed | N/A |
| SPRS | 28 | N/A |

UL – upper limb, LL – lower limb, M – male, F – female, Vibr/Prop/Temp/Noci impairment of vibration sense/proprioception/temperature sensation/nociception, CMCT ms central motor conduction time in milliseconds, N/A not available. Reflex grading: 0 absent, + reduced, ++ normal, +++ brisk without clonus, ++++ brisk with clonus. *Previously reported by Kumar *et al* ^15^. ^#^Previously reported in Abrahamsen *et al*^10^.
